# Supplementary material for: Structure of the turnover-ready state of an ancestral respiratory complex I
Source: Nat Commun. 2024 Oct 29;15:9340. doi: 10.1038/s41467-024-53679-3 (PMC11522691; doi:10.1038/s41467-024-53679-3)
Supplement: Supplementary file 1 — Supplementary Information [file 41467_2024_53679_MOESM1_ESM.pdf]

Supplementary Information

Structure of the turnover-ready state of an ancestral respiratory complex I

Bozhidar S. Ivanov[1], Hannah R. Bridges[1,2], Owen D. Jarman[1,3] and Judy Hirst[1]*

[1]The Medical Research Council Mitochondrial Biology Unit, University of Cambridge, Keith Peters Building, Cambridge Biomedical Campus, Cambridge, United Kingdom

[2]Curent address: Structura Biotechnology Inc., Toronto, Canada

[3]Current address: Department of Biochemistry and Synthetic Metabolism, Max Planck Institute for Terrestrial Microbiology, Marburg, Germany

*e-mail: jh480@cam.ac.uk

This file includes:

Supplementary Figs. 1 to 11

Supplementary Tables 1 to 4

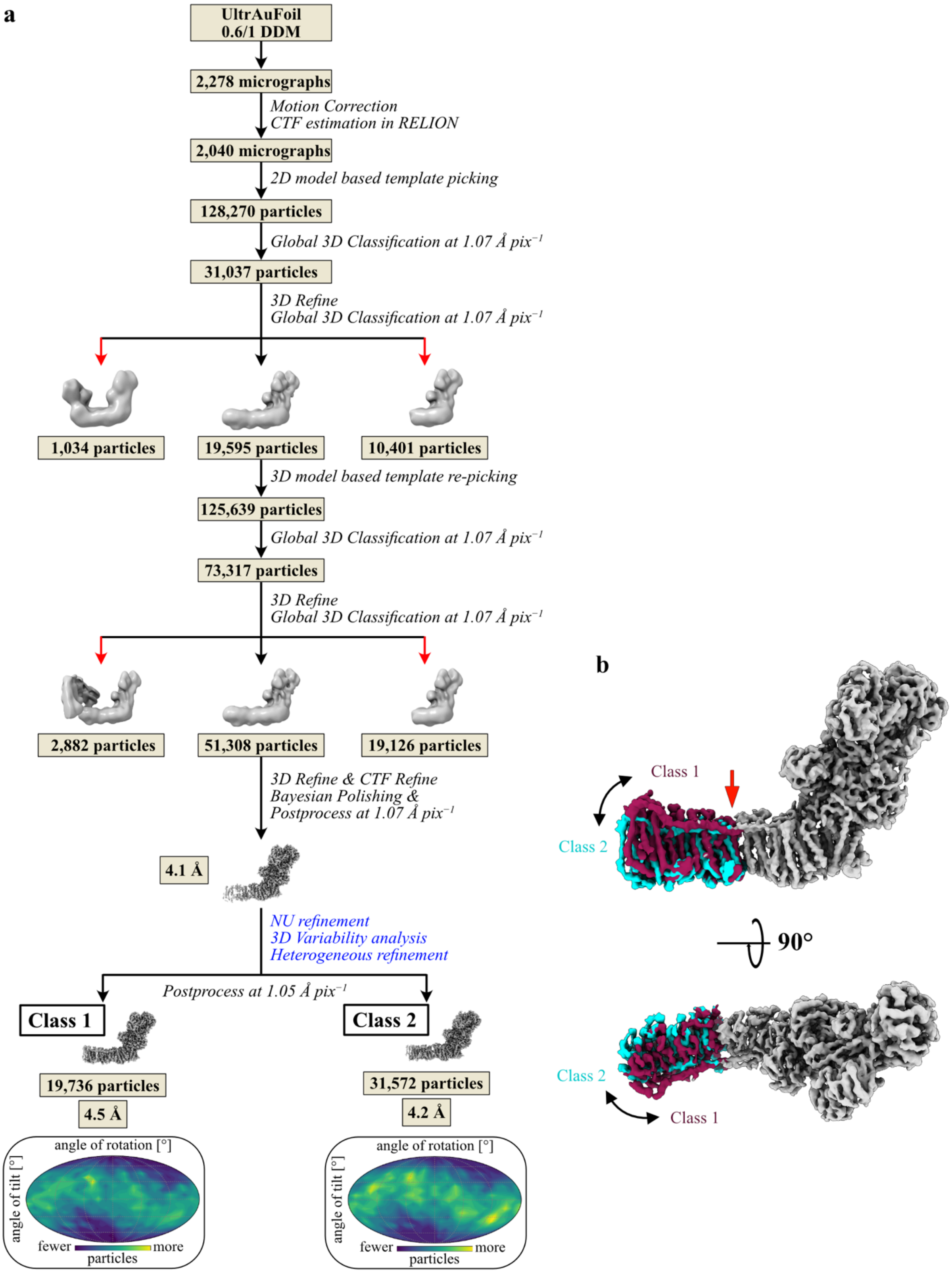

Supplementary Figure 1. Cryo-EM data processing pipeline for *Pd*-CI-DDM and comparison of classes 1 and 2. a) Cryo-EM data processing pipeline, with the red arrows indicating particles that were excluded. The orientation distributions for each class are shown. b) Comparison of the density maps for classes 1 and 2 aligned on the hydrophilic domain showing the different global conformations of the membrane domain. The cleft that opens up in class 2 between subunits ND2 and ND4, allowing the ND4-5 distal subdomain to move relative to the rest of the enzyme, is marked with an arrow. Processing steps in blue were carried out in *CryoSPARC v3.3.2*.

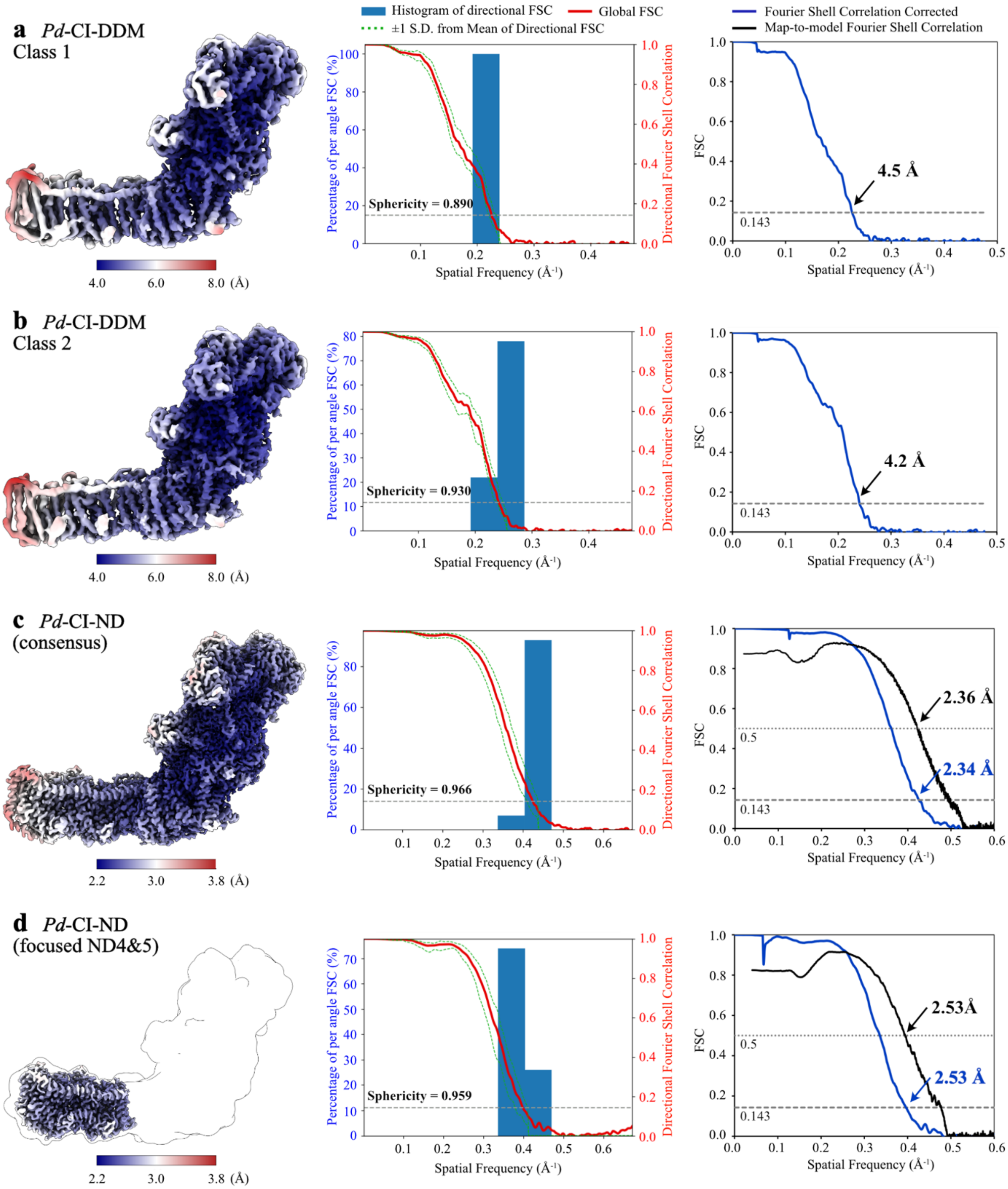

Supplementary Figure 2. Local resolution and FSC curves for all *Pd*-CI maps. Local resolution plots calculated in RELION and visualised in UCSF ChimeraX (left) are shown with 3D FSC curves and histograms showing the distribution of resolution shells (middle) and half-map/map-to-model FSC curves (right) for a) *Pd*-CI-DDM class 1; b) *Pd*-CI-DDM class 2; c) *Pd*-CI-ND (consensus); and d) *Pd*-CI-ND focussed map for the distal membrane subdomain.

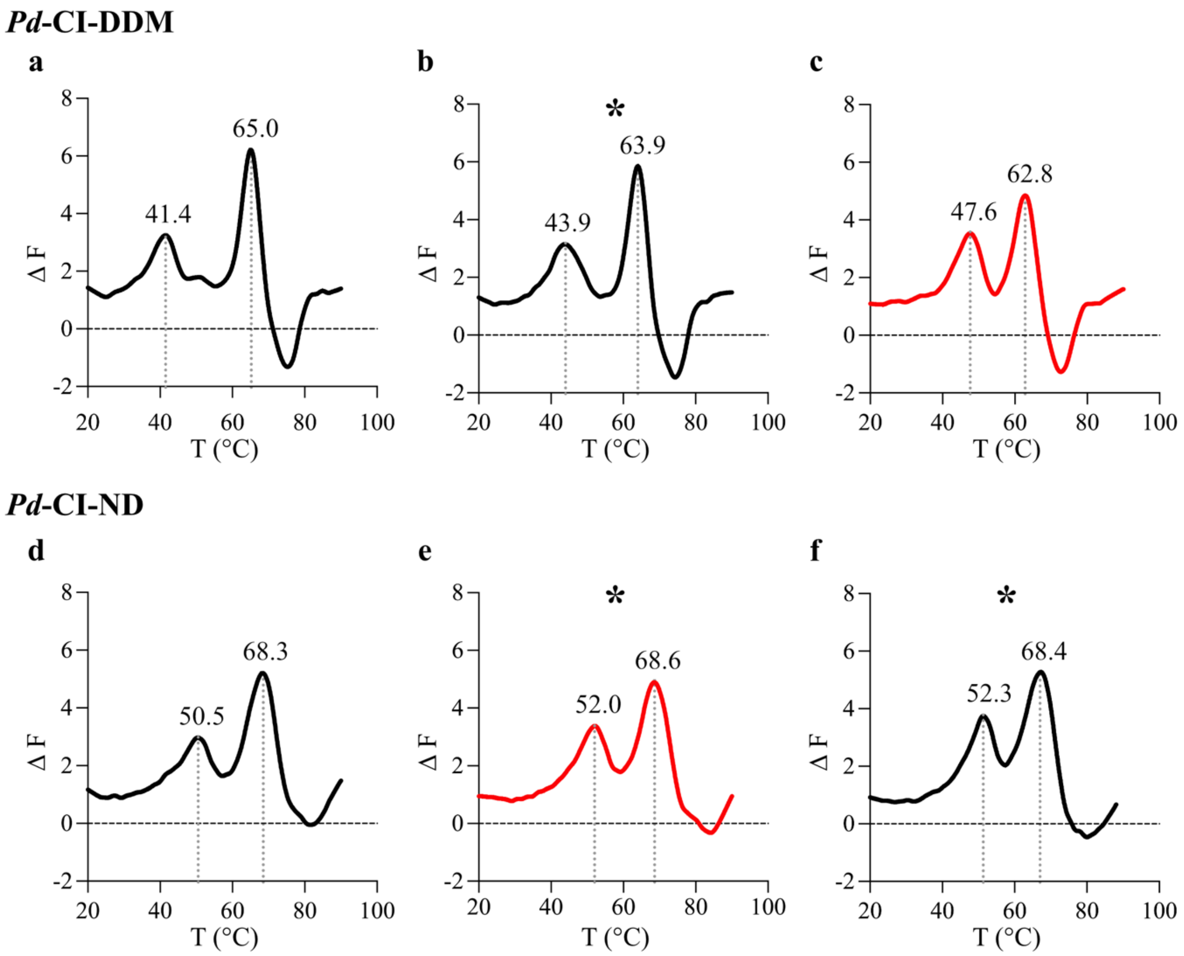

Supplementary Figure 3. Comparison of the stability of *Pd*-CI-DDM and *Pd*-CI-ND preparations by nano-DSF. The data are presented as the first derivative of the change in fluorescence as the temperature is increased with peak positions labelled. a) *Pd*-CI-DDM in the buffer used for size-exclusion chromatography (20 mM MES (pH 6.5 at 4 °C), 150 mM NaCl, 10 mM $CaCl_2$, 10% (v/v) glycerol, 0.05% (w/v) DDM). b) *Pd*-CI-DDM in the buffer for cryo-EM grid preparation (the buffer from (a) but without glycerol). c) *Pd*-CI-DDM in 20 mM MES (pH 6.5 at 4 °C), 25 mM NaCl, 1 mM $CaCl_2$ and 0.05% DDM. d) *Pd*-CI-ND in ND reconstitution buffer (20 mM MES (pH 6.5 at 4 °C) and 25 mM NaCl). e) *Pd*-CI-ND in the buffer for cryo-EM grid preparation (ND reconstitution buffer plus 1 mM $CaCl_2$). f) *Pd*-CI-ND with FOM in the buffer for cryo-EM grid preparation (ND reconstitution buffer plus 1 mM $CaCl_2$ and 0.01% FOM). Panels (c) and (e) (red) directly compare *Pd*-CI-DDM and *Pd*-CI-ND under matching conditions, and asterisks denote conditions that were used to make cryo-EM grids. Each trace was repeated three times, and the peak positions were consistent within 0.5 °C ranges. Source data are provided as a Source Data file.

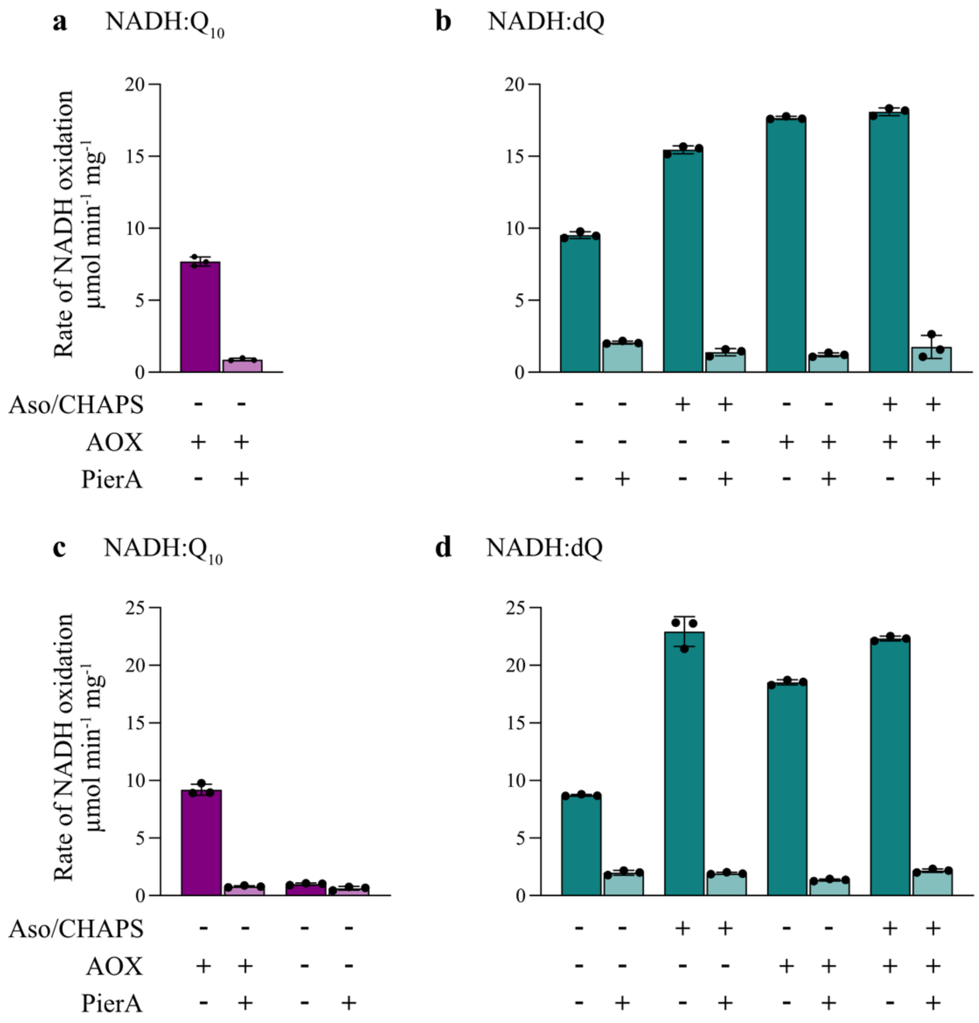

Supplementary Figure 4. Catalytic activities of the two *Pd*-CI-ND preparations analyzed by cryo-EM. a) Rates of NADH:ubiquinone-10 oxidoreduction using the ubiquinone-10 contained in the nanodiscs and recycled by AOX. b) Rate of NADH:dQ oxidoreduction by *Pd*-CI-ND (grids 1 and 2). Assays were carried out using 200 µM dQ as described in *Materials and Methods*, with the addition of asolectin and CHAPS to solubilise the nanodiscs, AOX to recycle ubiquinol, and piericidin A to inhibit catalysis as indicated. c) and d) Equivalent data for *Pd*-CI-ND (grid 3) frozen in the presence of FOM. All rates have been normalised for the complex I content determined using the NADH:APAD$^+$ oxidoreduction assay. The activities of the two *Pd*-CI-DDM preparations before reconstitution were 22.0 ± 1.9 (grids 1 and 2) and 19.5 ± 0.2 µmol min^{-1} mg^{-1} (grid 3) (208 and 184 s^{-1}, respectively). The units can be converted using a factor of 9.45. Source data are provided as a Source Data file.

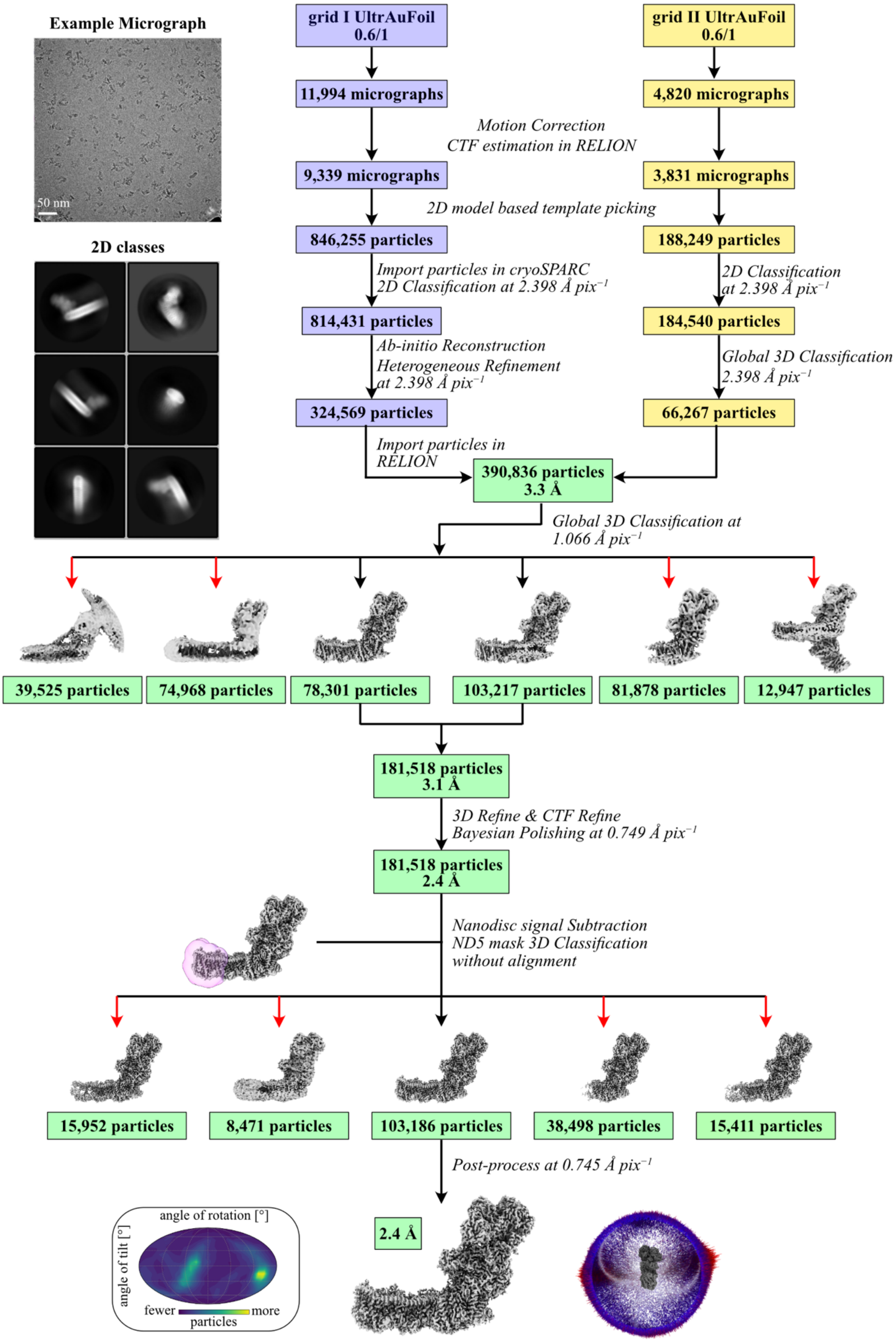

Supplementary Figure 5. Cryo-EM data processing pipeline for grids 1 and 2 for *Pd*-CI-ND. The red arrows indicate particles that were excluded, and the orientation distributions are shown.

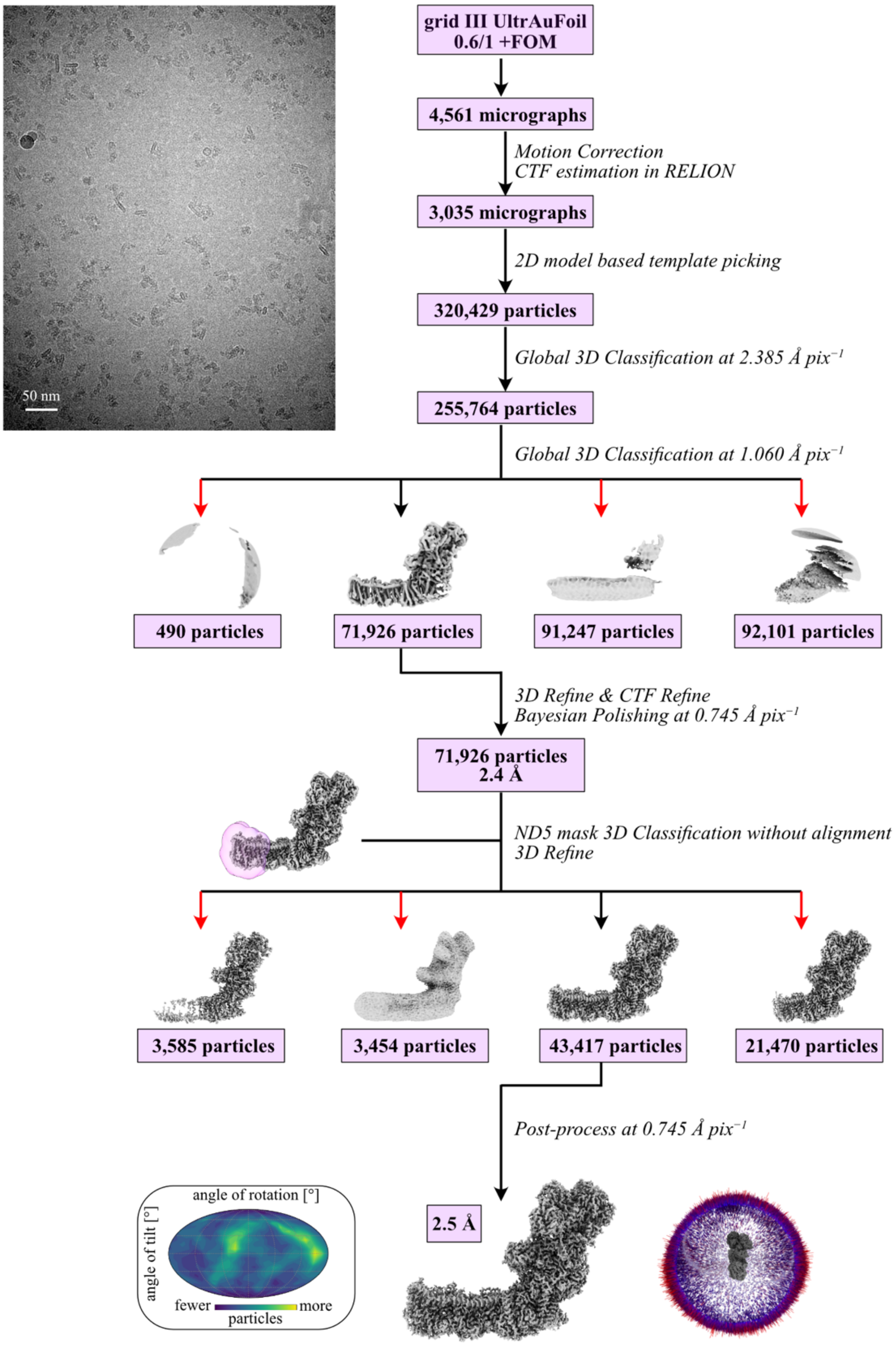

Supplementary Figure 6. Cryo-EM data processing pipeline for grid 3 for *Pd*-CI-ND in the presence of FOM. The red arrows indicate particles that were excluded, and the orientation distributions are shown.

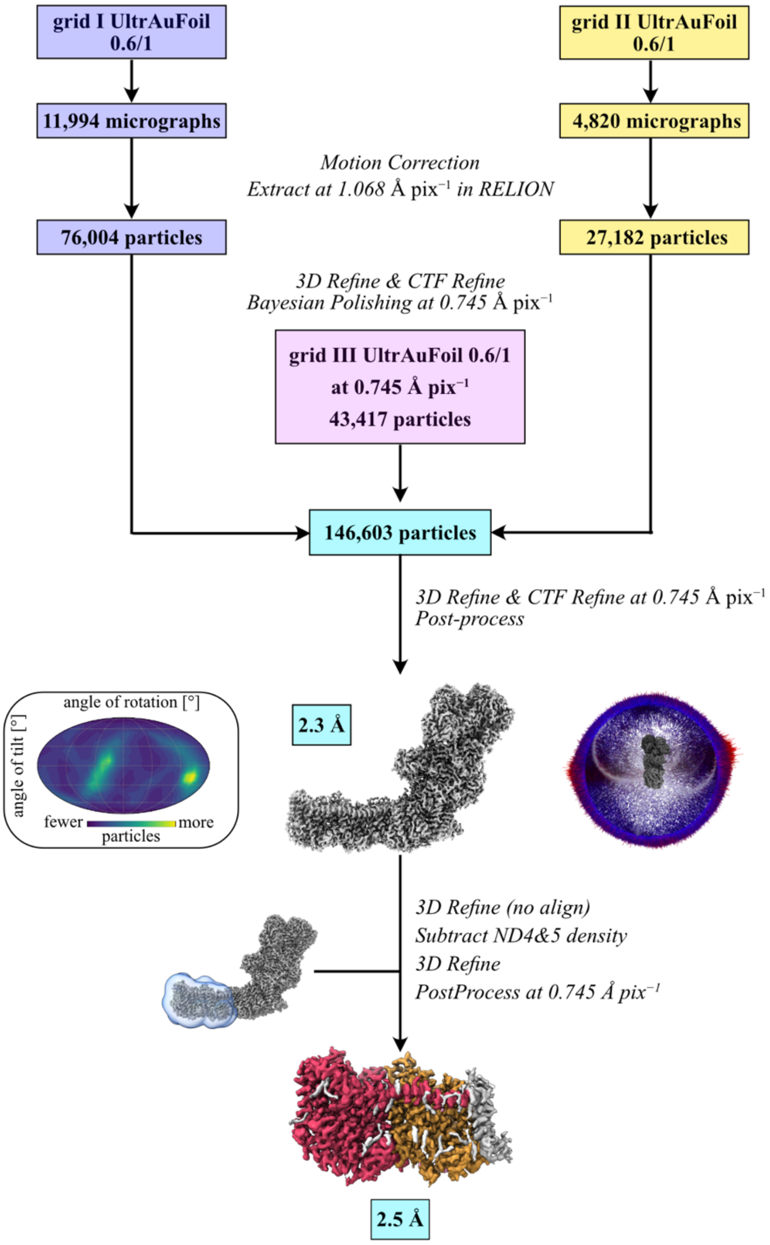

Supplementary Figure 7. The processing pipeline to combine data from cryo-EM grids 1, 2 and 3 for *Pd*-CI-ND, and the focussed refinement of the distal membrane subdomain. The distal section contains subunits ND4 (orange) and ND5 (red).

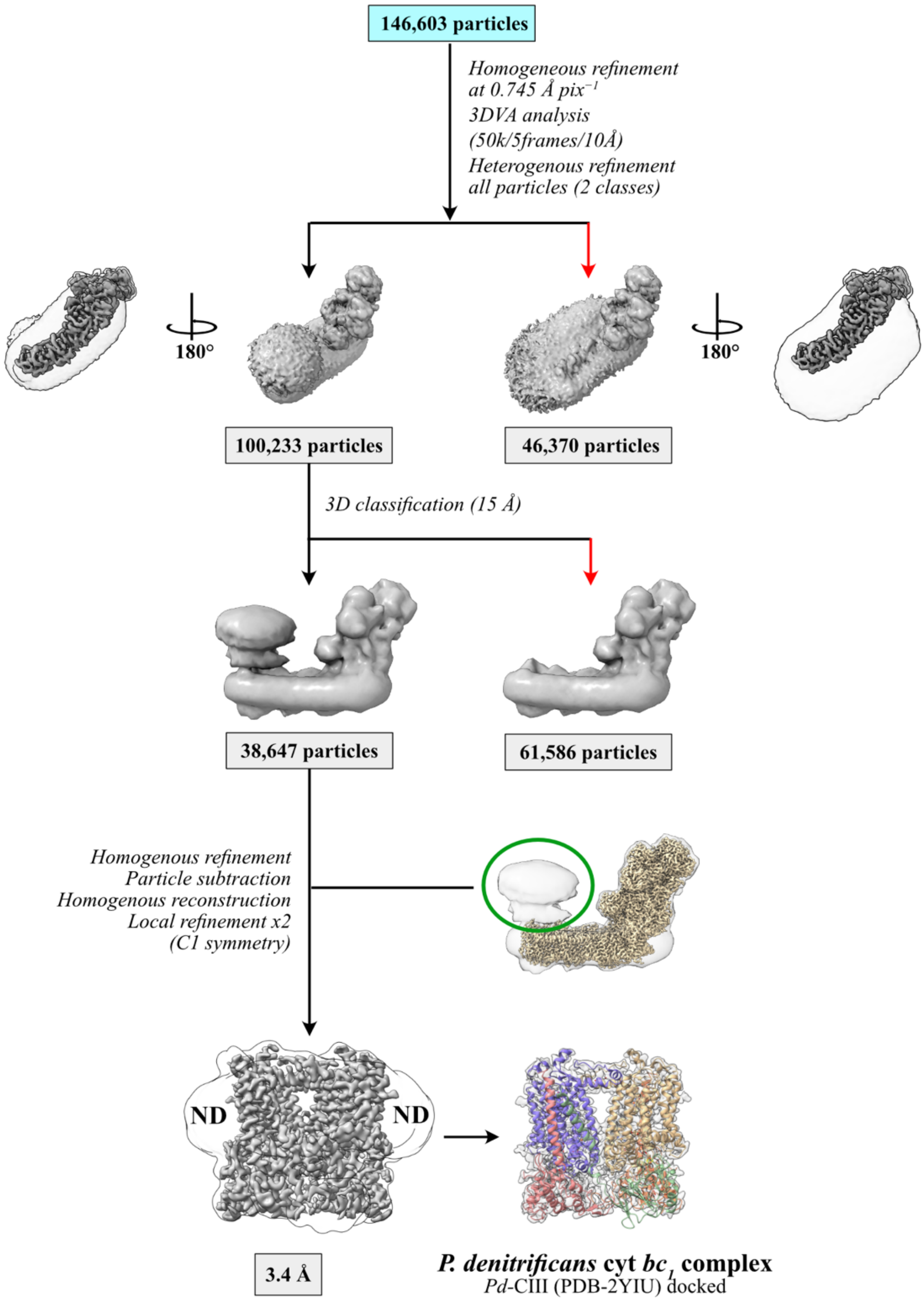

Supplementary Figure 8. Identification of two nanodisc populations and subclassification to identify the *bc*$_1$ complex. Small and large NDs are indicated in solid (top-view) and transparent (bottom-view) densities. The red arrows indicate excluded classes. The structure of the *P. denitrificans* cyt *bc*$_1$ complex PDB-2YIU is shown fitted into the 3.4-Å density map (EMDB: 19977).

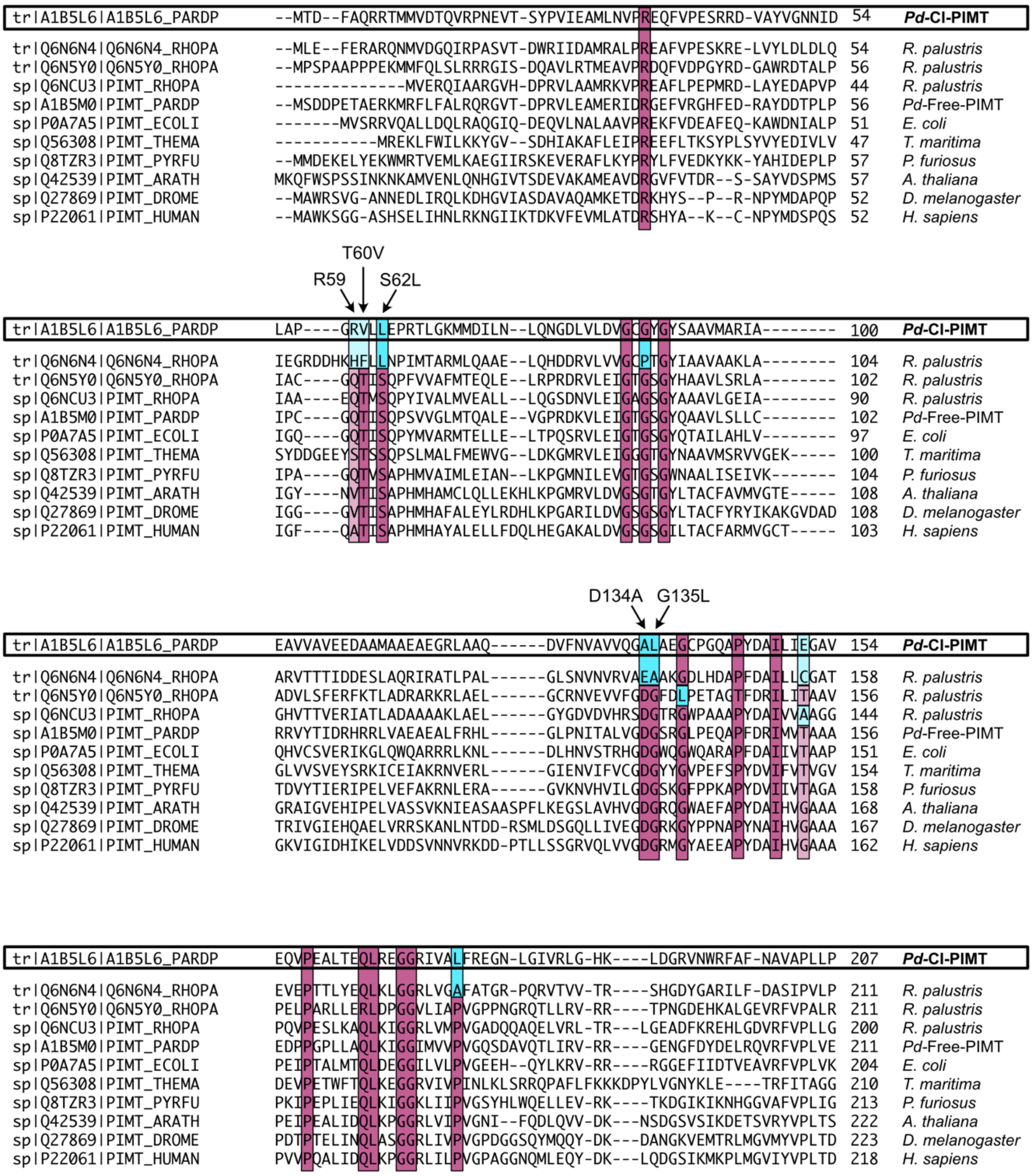

Supplementary Figure 9. Sequence alignment of PIMT across different species. Figure showing the sequence alignment of *Pd*-CI-PIMT (A1B5L6) against the free PIMT paralog from *Pd*-CI (A1B5M0) and orthologues from *Rhodopseudomonas palustris* (Q6N6N4, Q6N5Y0, Q6NCU3), *Escherichia coli* (P0A7A5)*, Thermotoga maritima* (Q56308)*, Pyrococcus furiosus* (Q8TZR3)*, Arabidopsis thaliana* (Q42539), *Drosophila melanogaster* (Q27869) *and Homo sapiens* (P22061). Universally conserved residues are highlighted in maroon while divergent mutations in *Pd*-CI-PIMT and *R. palustris* Q6N6N4 are shown in cyan. Lighter shades indicate small side-chain amino acids residues that are mutated to bulkier residues in *Pd*-CI-PIMT to fill the space occupied by the permanently bound SAM cofactor in functional PIMTs.

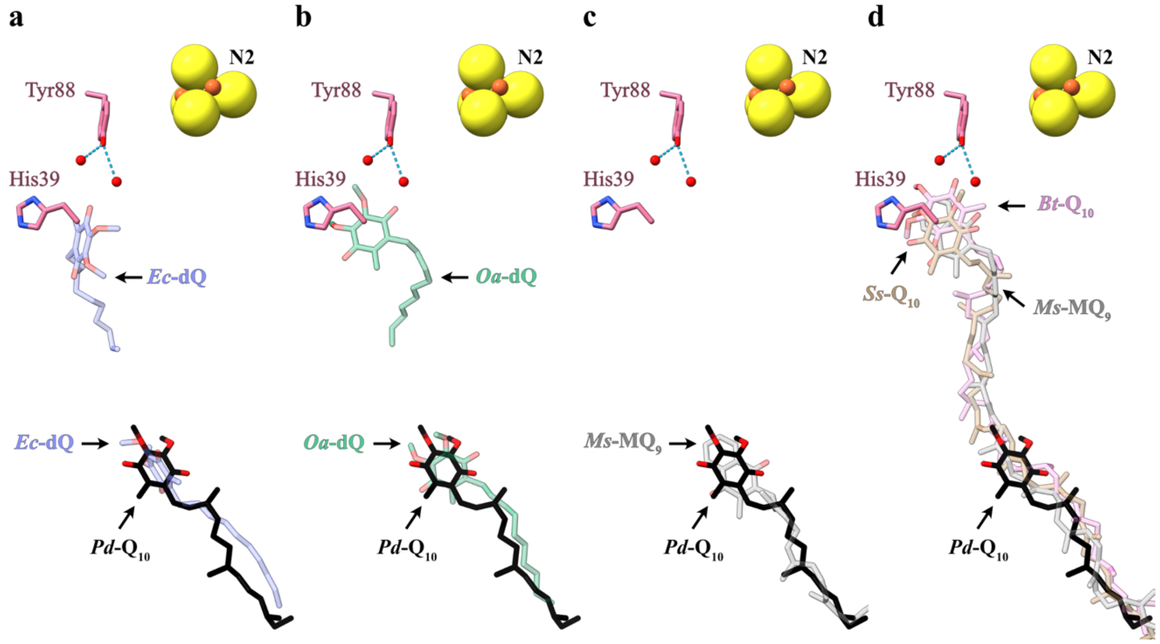

Supplementary Figure 10. Comparison of closed structures with Q_{10}, menaquinone-9 (MQ_9) and short-chain decylubiquinone (dQ) bound to the Q_{10}-bound structure of *Pd*-CI. a) The dQ bound in *Ec*-CI (blue, PDB-7Z7S) overlayed with the partially bound Q_{10} in *Pd*-CI. b) Comparison of the fully and partially inserted dQ molecules in ovine CI (green, PDB-6ZKC) with the Q_{10} bound in *Pd*-CI. c) Partially bound MQ_9 in *M. smegmatis* CI (grey, PDB-8E9G) overlayed with partially bound Q_{10} in *Pd*-CI. d) Fully inserted Q_{10} or MQ_9 positions in bovine (pink, PDB-7QSK), porcine (wheat, PDB-7V2C) and *M. smegmatis* complex I showing the ~20 Å distance between the two Q head groups binding positions.

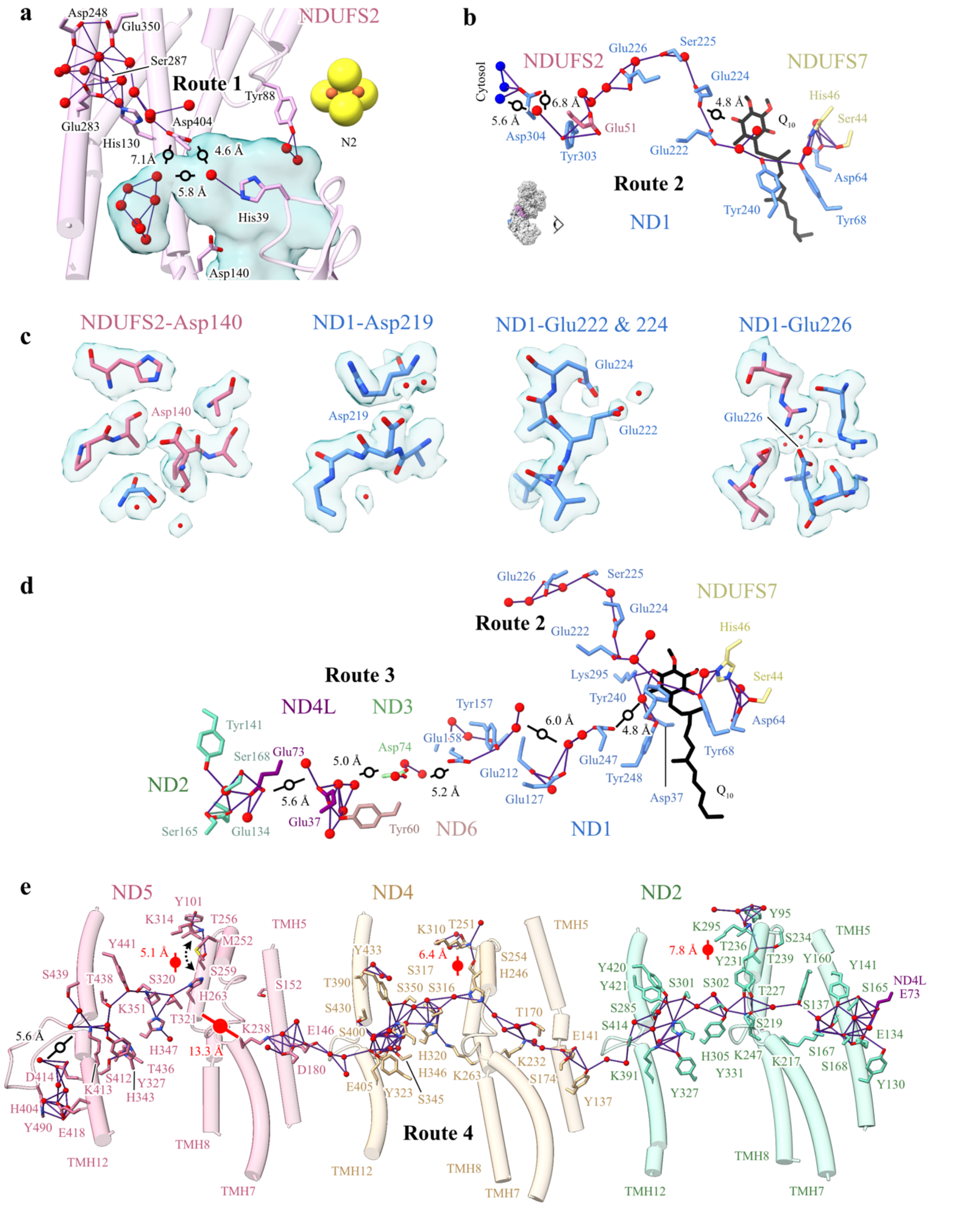

Supplementary Figure 11. Grotthuss competent pathways in *P. denitrificans* complex I at the ubiquinone site, E-channel, and central axis. a) A potential ubiquinone-10 protonation pathway leading to the active site (route 1). b) A second potential ubiquinone-10 protonation pathway leading to the secondary Q-binding site (route 2). c) The locally sharpened cryo-EM densities of key acidic residues (cryo-EM density outlines at a 1.5 Å range from the displayed atoms are shown as a semi-transparent surface in UCSF ChimeraX at map threshold of 0.01) with residues or water molecules within 3 Å distance. d) Grotthuss competent network of the lower section of the ubiquinone-10 binding site and the E-channel (routes 2 and 3). e) Grotthuss competent networks in the ND2, ND4 and ND5 subunits in *Pd*-CI (route 4). The networks are shown with purple lines and the gaps are indicated by black lines (with distances displayed) and open circles highlighting protein unobstructed routes. Red lines with full circles indicate protein obstructed gaps.

Supplementary Table 1. Cryo-EM data collection, refinement, and validation statistics for *Pd*-CI-DDM.

Data collection and processing	*Pd*-CI-DDM	
Voltage (kV)	300	
Nominal magnification	130,000×	
Electron exposure (e^{-} Å^{-2})	48	
Defocus range (µm)	-1.5 to -2.9	
Calibrated pixel size (Å)	1.05	
Number of frames	25	
Energy filter slit width (eV)	20	
Symmetry imposed	C1	
Number of micrographs	2,278	
Initial particle images (no.)	128,270	
Final particle images (no.)	51,308	
Final Global Classification	Class 1 (EMDB: 19975)	Class 2 (EMDB: 19976)
Final particle images (no.)	19,736	31,572
Map sharpening *B* factor (Å^2)	-76.26	-95.39
Map resolution (Å) (FSC = 0.143)	4.5	4.2
Map resolution range (Å)	3.75 – 12.65	3.99 – 12.55

Supplementary Table 2. Summary of the model for *Pd*-CI-ND (PDB-8QBY).

Pd-CI subunit	Human CI subunit	Chain identity	Total residues	Modelled residues (%)	Q-score	Cofactors & modification
Nqo1	NDUFV1	F	431	1-421 (97.7)	0.83	FMN, 4Fe4S
Nqo2	NDUFV2	E	239	1-236 (98.7)	0.83	2Fe2S
Nqo3	NDUFS1	G	674	2-667 (98.8)	0.84	2Fe2S, 2 x 4Fe4S, Na^{+}
Nqo4	NDUFS2	D	412	3-412 (99.5)	0.87	Dimethyl-Arg65, Ca^{2+}
Nqo5	NDUFS3	C	208	6-196 (91.8)	0.86	
Nqo6	NDUFS7	B	175	28-175 (84.6)	0.87	4Fe4S
Nqo9	NDUFS8	I	163	3-163 (98.8)	0.87	2 x 4Fe4S
Nqo8	ND1	H	345	1-342 (99.1)	0.86	
Nqo14	ND2	N	499	1-479 (96.0)	0.86	Ca^{2+}
Nqo7	ND3	A	121	1-121 (100)	0.86	N-formyl
Nqo13	ND4	M	513	1-503 (98.1)	0.83, 0.83[2]	Ca^{2+}
Nqo11	ND4L	K	101	1-101 (100)	0.86	
Nqo12	ND5	L	703	1-506, 551-703 (93.7)	0.78, 0.82[2]	
Nqo10	ND6	J	200[1]	1-82, 88-200 (97.5)	0.84	N-terminus 2 x Met
*Pd*NUYM	NDUFS4	Q	103	1-103 (100)	0.84	
*Pd*NUMM	NDUFS6	R	62	3-61 (95.2)	0.83	Zn^{2+}, Ca^{2+}
*Pd*N7BM	NDUFA12	q	124	1-124 (100)	0.84	
PIMT	-	t	217	2-217 (99.5)	0.83	

[1]The total number of residues for ND6 is 200, taking into account the two Met residues observed at the N-terminus

[2]Q-score determined for the focused map of the membrane ND4-ND5 subdomain

Supplementary Table 3. Sequence and structural comparison of *Pd*-CI to other CI species.

Subunit	Relative to *B. taurus* complex I subunits				
	Sequence identity (%)	Length (%) (N of residues)	RMSD[1] values (Å)		
			B. taurus	*Y. lipolytica*	*E. coli*
NDUFV1 (Nqo1)	64.0	92.9 (-33)	0.60	0.89	2.34
NDUFV2 (Nqo2)	40.2	96.0 (-10)	6.66	9.00	1.35
NDUFS1 (Nqo3)	47.5	92.7 (-53)	3.14	2.25	11.68
NDUFS1 N/C-domains	61.9/40.5	92.7 (-18/-35)	1.07/3.89	1.11/2.55	3.49/13.97
NDUFS2 (Nqo4)	59.0	89.0 (-51)	5.17	5.69	2.72
NDUFS3 (Nqo5)	46.9	78.2 (-58)	1.97	1.51	19.89
NDUFS7 (Nqo6)	68.0	81.0 (-41)	1.13	1.43	1.41
NDUFS8 (Nqo9)	73.0	76.9 (-49)	0.80	1.07	3.20
ND1 (Nqo8)	39.1	108.5 (+27)	2.51	3.25	1.81
ND2 (Nqo14)	25.7	143.8 (+152)	4.51	3.75	3.31
ND3 (Nqo7)	37.5	105.2 (+6)	1.31	3.57	2.30
ND4 (Nqo13)	31.4	111.8 (+54)	3.01	4.81	2.32
ND4L (Nqo11)	22.9	103.1 (+3)	1.64	1.73	1.17
ND5 (Nqo12)	34.7	116.0 (+97)	4.09	3.48	4.05
ND6 (Nqo10)	23.6	114.3 (+25)	7.25	6.99	4.77
NDUFS4 (dNUYM)	36.0	58.9 (-72)	2.51	3.03	-
NDUFS6 (PdNUMM)	32.7	50.0 (-62)	2.21	4.27	-
NDUFA12 (PdN7BM)	37.0	85.5 (-21)	9.56	8.94	-
PIMT	-	-	-	-	-

[1]The structures used for the RMSD comparisons were PDB-7QSL (2.8 Å-resolution bovine structure in the closed state), PDB-6YG4 (2.7 Å-resolution *Y. lipolytica* structure) and PDB-7Z7S (2.4 Å-resolution *E. coli* structure in the closed state). RMSD values were calculated using UCSF ChimeraX including all pairs of residues.

Supplementary Table 4. Structural investigation of the inter-domain angle of complex I.

	Species	Given name	PDB	Interdomain angle[1] (°)		
				4Fe-ND1-ND5	4Fe-ND1-ND2	N2-ND1-ND4L
Bacteria	*Paracoccus denitrificans*	Active/closed	this work	117.0	124.2	127.5
	Mycobacterium smegmatis	Active	8E9G	118.3	126.0	128.8
	Escherichia coli	Closed	7Z7S	118.7	119.2	126.9
Single cell eukaryote	*Tetrahymena thermophila*	Closed	7TGH	112.4	121.1	130.7
Insect	*Drosophila melanogaster*	Active (closed)	8B9Z	113.1	120.5	127.6
Mammal	*Bos taurus*	Active-apo (closed)	7QSL	112.0	120.3	120.4
		Deactive-apo (open)	7QSN	113.5	121.2	125.3
				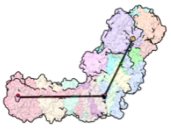	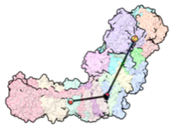	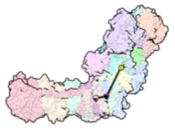

[1]The conserved reference points chosen to measure the inter-arm angles were the N2 [4Fe-4S] cluster in NDUFS7, the 4Fe all-Cys ligated [4Fe-4S] cluster in NDUFS1 and the Cα of *Pd*-ND1-L126, *Pd*-ND5-F349 and *Pd*-ND4L-E36. Angles were measured using UCSF ChimeraX. The structures used in this analysis were PDB-8E9G, PDB-7Z7S, PDB-7TGH, PDB-8B9Z, PDB-7QSL and PDB-7QSN.
