# Supplementary material for: Structure of the turnover-ready state of an ancestral respiratory complex I
Source: Nat Commun. 2024 Oct 29;15:9340. doi: 10.1038/s41467-024-53679-3 (PMC11522691; doi:10.1038/s41467-024-53679-3)
Supplement: Supplementary file 2 — Reporting Summary [file 41467_2024_53679_MOESM2_ESM.pdf]

nature portfolio

Corresponding author(s): Judy Hirst

Last updated by author(s): Oct 7, 2024

Reporting Summary

Nature Portfolio wishes to improve the reproducibility of the work that we publish. This form provides structure for consistency and transparency in reporting. For further information on Nature Portfolio policies, see our Editorial Policies and the Editorial Policy Checklist.

Statistics

For all statistical analyses, confirm that the following items are present in the figure legend, table legend, main text, or Methods section.

n/a	Confirmed	
☐	☒	The exact sample size (*n*) for each experimental group/condition, given as a discrete number and unit of measurement
☐	☒	A statement on whether measurements were taken from distinct samples or whether the same sample was measured repeatedly
☒	☐	The statistical test(s) used AND whether they are one- or two-sided<br>*Only common tests should be described solely by name; describe more complex techniques in the Methods section.*
☒	☐	A description of all covariates tested
☒	☐	A description of any assumptions or corrections, such as tests of normality and adjustment for multiple comparisons
☐	☒	A full description of the statistical parameters including central tendency (e.g. means) or other basic estimates (e.g. regression coefficient) AND variation (e.g. standard deviation) or associated estimates of uncertainty (e.g. confidence intervals)
☒	☐	For null hypothesis testing, the test statistic (e.g. *F*, *t*, *r*) with confidence intervals, effect sizes, degrees of freedom and *P* value noted<br>*Give P values as exact values whenever suitable.*
☒	☐	For Bayesian analysis, information on the choice of priors and Markov chain Monte Carlo settings
☒	☐	For hierarchical and complex designs, identification of the appropriate level for tests and full reporting of outcomes
☒	☐	Estimates of effect sizes (e.g. Cohen's *d*, Pearson's *r*), indicating how they were calculated

Our web collection on statistics for biologists contains articles on many of the points above.

Software and code

Policy information about availability of computer code

Data collection	Softmax Pro, EPU, Prometheus NT.48
Data analysis	RELION 3.0.7 and 3.1.0, GCTF v 1.18, CTFFIND-4.1, CryoSPARC 2.7 and 3.3.2, UCSF ChimeraX, ModelAngelo, Coot 0.9, Phenix v 1.20

For manuscripts utilizing custom algorithms or software that are central to the research but not yet described in published literature, software must be made available to editors and reviewers. We strongly encourage code deposition in a community repository (e.g. GitHub). See the Nature Portfolio guidelines for submitting code & software for further information.

Data

Policy information about availability of data

All manuscripts must include a data availability statement. This statement should provide the following information, where applicable:

- Accession codes, unique identifiers, or web links for publicly available datasets
- A description of any restrictions on data availability
- For clinical datasets or third party data, please ensure that the statement adheres to our policy

Structural data have been deposited in the EMDB and PDB databases under the following accession codes: EMD-18324 and PDB-8QBY (complex I-nanodiscs), EMD-18325 and PDB-8QC1 (ND4&5 focussed refined map & model), EMD-19975 (complex I-DDM Class 1) EMD-19976 (complex I-DDM Class 2), EMD-19977 (cytochrome bc1 complex). The cryo-EM raw images are available from EMPIAR with the accession codes EMPIAR-12077 (complex I-nanodiscs grids 1 and 2), EMPIAR-12078 (complex I-nanodiscs grid 3), and EMPIAR-12079 (complex I-DDM).

Research involving human participants, their data, or biological material

Policy information about studies with human participants or human data. See also policy information about sex, gender (identity/presentation), and sexual orientation and race, ethnicity and racism.

Reporting on sex and gender	
Reporting on race, ethnicity, or other socially relevant groupings	
Population characteristics	
Recruitment	
Ethics oversight	

Note that full information on the approval of the study protocol must also be provided in the manuscript.

Field-specific reporting

Please select the one below that is the best fit for your research. If you are not sure, read the appropriate sections before making your selection.

- [x] Life sciences
- [ ] Behavioural & social sciences
- [ ] Ecological, evolutionary & environmental sciences

For a reference copy of the document with all sections, see nature.com/documents/nr-reporting-summary-flat.pdf

Life sciences study design

All studies must disclose on these points even when the disclosure is negative.

Sample size	Cryo-EM data were measured for three samples originating from a three different protein purifications. Entire data sets contained 128,270, 1,034,504 and 320,429 particles picked, which were then filtered by 2D and 3D classification. Final classes contained a total of 51,308, 103,186 and 43,417 particles, respectively.
Data exclusions	After particle classification, data for non-protein or damaged protein images were removed (see Methods). No data were excluded from biochemical or computational experiments.
Replication	Cryo-EM data were measured on three different data sets. Kinetic measurements were of 3 technical replicates.
Randomization	N/A
Blinding	N/A

Reporting for specific materials, systems and methods

We require information from authors about some types of materials, experimental systems and methods used in many studies. Here, indicate whether each material, system or method listed is relevant to your study. If you are not sure if a list item applies to your research, read the appropriate section before selecting a response.

Materials & experimental systems

n/a	Involved in the study	
x		Antibodies
x		Eukaryotic cell lines
x		Palaeontology and archaeology
	x	Animals and other organisms
x		Clinical data
x		Dual use research of concern
x		Plants

Methods

n/a	Involved in the study	
x		ChIP-seq
x		Flow cytometry
x		MRI-based neuroimaging

Animals and other research organisms

Policy information about studies involving animals; ARRIVE guidelines recommended for reporting animal research, and Sex and Gender in Research

Laboratory animals	Paracoccus denitrificans Pd1222-ΔHy-Nqo5His6
Wild animals	
Reporting on sex	
Field-collected samples	
Ethics oversight	

Note that full information on the approval of the study protocol must also be provided in the manuscript.

Plants

Seed stocks	
Novel plant genotypes	
Authentication	
